# Editorial for the Special Issue on Sustainable Materials for Energy and Environmental Applications

**DOI:** 10.3390/mi15091099

**Published:** 2024-08-30

**Authors:** Arun Thirumurugan, Sathish Kumar Kamaraj, Udayabhaskar Rednam, Ramesh Raju

**Affiliations:** 1 Sede Vallenar, Universidad de Atacama, Vallenar 1612178, Chile; 2 Instituto Politécnico Nacional (IPN)-Centro de Investigación en Ciencia Aplicada y Tecnología Avanzada (CICATA-Altamira), Carretera Tampico-Puerto Industrial Altamira Km14.5, C. Manzano, Industrial Altamira, Altamira 89600, Tamaulipas, Mexico; sathish.k@llano.tecnm.mx; 3 Departamento de Mecánica, Facultad de Ingeniería, Universidad Tecnológica Metropolitana, Santiago 7800003, Chile; uday.rednam@uda.cl; 4 Department of Electrical and Nanoengineering, Aalto University, Tietotie 3, 02150 Espoo, Finland; ramesh.raju@aalto.fi

Sustainable environmental management is an urgent problem that calls for innovative solutions to the problem of protecting and preserving the natural resources and ecosystems of the planet [1,2,3,4]. Our global community is currently grappling with the urgent requirement to tackle issues such as climate change, environmental degradation, and resource shortages. As a result, advancements in environmentally friendly materials and technologies are emerging as a result of efforts to establish a more resilient and sustainable future. It is the primary objective of this Special Issue on “Sustainable Materials for Energy and Environmental Applications” to shed light on the most recent developments in this important field. We also aim to demonstrate the future promise of cutting-edge research in materials science for advancing sustainable energy production, environmental remediation, and resource conservation. This Special Issue brings together a wide variety of research and review articles that investigate the synthesis, characterization, and application of a wide range of nanomaterials. These nanomaterials include metals, metal oxides, 2D materials, carbon-based materials, and their composites. These nanomaterials are developed using environmentally friendly and sustainable methods. Not only do our contributions exhibit substantial advances in the field, but they also emphasize the interdisciplinary nature of research on sustainable materials. These contributions encompass a variety of fields, including agriculture, energy production, environmental science, biotechnology, and materials science.

Among the research articles featured, Ji Myeong Lee et al. present a study on “Amine-Impregnated Dendritic Mesoporous Silica for the Adsorption of Formaldehyde”, which addresses the challenge of indoor air pollution by developing a highly effective adsorbent for harmful volatile organic compounds [5]. The findings of this study highlight the significance of the pore structure in the process of improving adsorption capacities. These findings provide useful insights into the design of materials applicable to environmental remediation. Mohamed Rabia et al. contribute to the growing body of research on sustainable energy with their article, “Cr_2_S_3_-Cr_2_O_3_/Poly-2-aminobenzene-1-thiol as a Highly Photocatalytic Material for Green Hydrogen Generation from Sewage Water [6]”. Their cutting-edge nanocomposite photoelectrode demonstrates remarkable photon absorption and hydrogen generation efficiency, thereby paving the way for green hydrogen to become a viable alternative to fossil fuels.

In the field of photocatalysis, Sakthivel Kumaravel et al. have published a study titled “Highly Efficient Solar-Light-Active Ag-Decorated g-C_3_N_4_ Composite Photocatalysts for the Degradation of Methyl Orange Dye [7]”. As a result of their work, the amazing potential of Ag/g-C_3_N_4_ composites for environmental purification has been demonstrated. These composites achieve excellent degrading efficiency and stability when exposed to solar light. An additional illustration of using environmentally friendly materials in practical applications is provided by Prabavathi Munirathinam et al. in their research paper titled “Self-Powered Triboelectric Nanogenerator for Security Applications [8]”. This study demonstrates the adaptability of triboelectric nanogenerators in terms of energy harvesting and security, highlighting the potential for environmentally friendly materials to improve technologies that are used in everyday life. The research paper entitled “ZnO/Graphene Composite from Solvent-Exfoliated Few-Layer Graphene Nanosheets for Photocatalytic Dye Degradation under Sunlight Irradiation” by Vasanthi Venkidusamy et al. focuses on the positive effects that composite materials have on the environment [9]. The results of their research demonstrate that ZnO/graphene nanocomposites are effective in photocatalytic applications, which presents a viable strategy for the purification of water and the remediation of water and environmental contaminants.

Not only does this Special Issue contain valuable research, but it also includes two in-depth evaluations that enhance our understanding of environmentally friendly materials. Mariana Martínez-Castrejón et al. conducted a comprehensive assessment of “Environmental, Economic, and Social Aspects of Human Urine Valorisation through Microbial Fuel Cells from the Circular Economy Perspective [10]”. The purpose of this review was to investigate the possible advantages of bioelectrochemical systems for resource recovery, with a particular focus on the significance of incorporating sustainable practices into waste management procedures.

Arun Thirumurugan et al. present a comprehensive study of “MXene/Ferrite Magnetic Nanocomposites for Electrochemical Supercapacitor Applications [11]”. In this study, the synthesis, characteristics, and electrochemical performance of composites based on MXene are discussed. Particular attention is paid to the potential of these composites for energy storage applications, as well as the exciting possibilities afforded by magnetic field-assisted supercapacitors.

The papers that are included in this Special Issue highlight the significant role that sustainable materials play in addressing global concerns associated with the sustainability of energy and the natural environment. Through advancements in research and the implementation of new materials, we are closer to the realization of a future in which environmentally responsible practices are at the forefront of energy generation, environmental protection, and resource management all at the same time. With the help of this collection of publications, we aim to encourage additional research and innovation in this extremely important sector, which will ultimately lead to a world that is more sustainable and equitable.
